# Supplementary material for: Dissecting Tumor Heterogeneity by Liquid Biopsy—A Comparative Analysis of Post-Mortem Tissue and Pre-Mortem Liquid Biopsies in Solid Neoplasias
Source: Int J Mol Sci. 2025 Aug 6;26(15):7614. doi: 10.3390/ijms26157614 (PMC12347833; doi:10.3390/ijms26157614)
Supplement: Supplementary file 1 [file ijms-26-07614-s001.zip › ijms-3790234-supplementary.pdf]

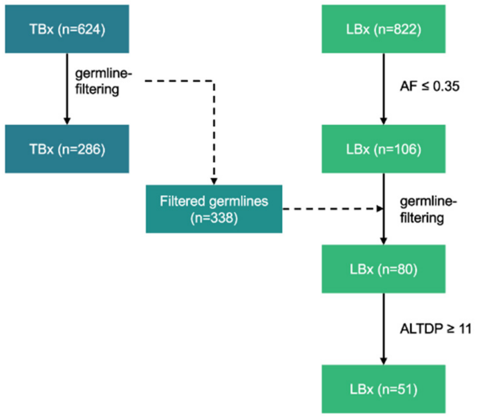

Supplemental Figure S1: Process of filtering TBx and LBx variants

Tissue variants (n=624) were filtered for germline alterations (blue). Filtering steps for LBx are shown in green. Dashed arrows indicate where the excluded germline tissue variants were used to filter liquid variants.

A

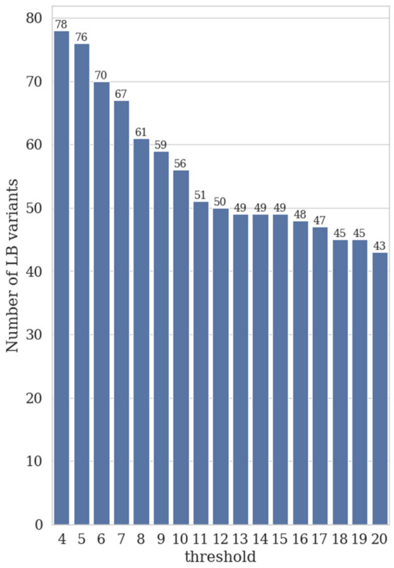

B

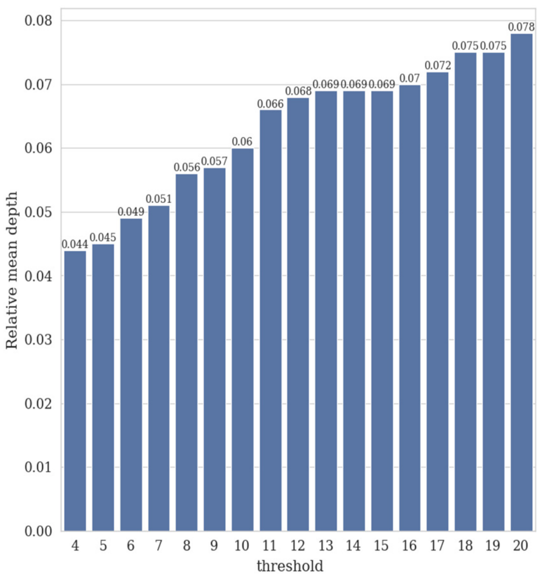

Supplemental Figure S2: Threshold analysis for filtering LBx variants

A: The barplot depicts the number of LBx variants (y axis) when applying different allele depth (AD) thresholds ranging from 4 to 20 (x axis).
B: Barplot representing the relative mean depth (y axis) when applying the corresponding AD threshold (4 – 20; x axis) in the filtering process

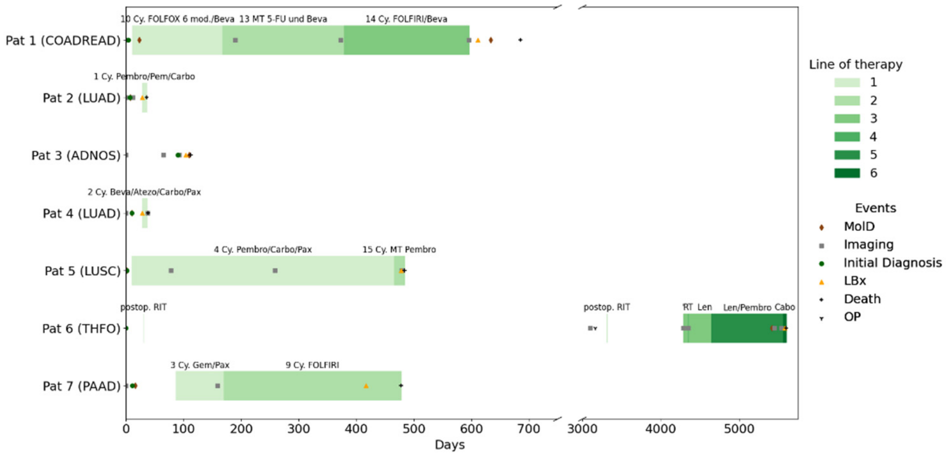

Supplemental Figure S3: Patient journeys over time

Patient journeys for the seven patients included in our study (y axis) over time (x axis).
Lines of therapy are shown in green, with the corresponding treatments described above each bar and different events represented by symbols (molecular diagnostics by a brown diamond, imaging by a gray square, initial diagnosis by a dark green circle, liquid biopsy by a yellow triangle, surgery by a black triangle, and death by a black plus sign).

COADREAD: Colorectal adenocarcinoma; LUAD: Lung adenocarcinoma; ADNOS: Adenocarcinoma not other specified; LUSC: Lung squamous cell carcinoma; THFO: Follicular thyroid cancer; PAAD: Pancreatic adenocarcinoma; Cy: Cycle; Atezo: Atezolizumab; Beva: Bevacizumab; 5-FU: 5-Fluorouracil; /Pem: Pemetrexed; Pembro: Pembrolizumab; Carbo: Carboplatin, Pax: Paclitaxel; Gem: Gemcitabine. Len: Lenvatinib, Cabo: Cabozantinib; postop.: postoperative; OP: Operation, MT: Maintenance therapy; LBx: Liquid biopsy; MolD: Molecular diagnostics

	**Patient**						
**Tissue**	**1 (COADREAD)**	**2 (LUAD)**	**3 (ADNOS)**	**4 (LUAD)**	**5 (LUSC)**	**6 (THFO)**	**7 (PAAD)**
**# 1**	Liver	Lobus superior pulmonis dextri	Pancreas/ local lymph nodes	Lobus inferior pulmonis sinistri	Lobus superior pulmonis sinistri	Lobus medius pulmonis dextri	Lobus inferior pulmonis dextri
**# 2**	Lobus inferior pulmonis sinistri	Liver	Pancreas/ local lymph nodes	Liver	Lobus superior pulmonis sinistri	Liver	Lobus superior pulmonis sinistri
**# 3**	Lobus superior pulmonis dextri	Liver	Pancreas/ local lymph nodes	Liver	Lobus superior pulmonis sinistri	Liver	Right liver lobe
**# 4**	Lobus medius pulmonis dextri	Liver	Pleura	Right adrenal gland	Lobus superior pulmonis sinistri	Cerebrum	Left liver lobe
**# 5**	Lobus inferior pulmonis dextri	Paratracheal Lymph node	Retroperitoneal lymph node	Left adrenal gland	Lobus superior pulmonis sinistri	Cerebellum	Kidney
**# 6**	Mesenterial lymph nodes	Spleen	Epicardial tissue	Rib	Left kidney	Right adrenal gland	Caput pancreatis
**# 7**	Peritoneal carcinomatosis	Pleura	Bone	Mediastinal lymph node	Right kidney	Mesenteric Tissue	Caput pancreatis
**# 8**	Colon	Paraaortal Tissue	Embolic metastasis (Lung)	Mediastinal lymph node	Prostate	Rib	Peripancreatic lymph nodes

Progress / new | intra-lesional samples
Stable
Regress
Not mentioned

Supplemental Figure S4: Tissue sample location and radiological findings

Tissue samples´ origins are shown per patient in columns. The response to treatment based on the previous last imaging is shown in red for progression/new location, yellow for stable, green for regression and white if not mentioned. Samples obtained from the same lesion (intra-lesional samples) are bordered in bold.
COADREAD: Colorectal adenocarcinoma; LUAD: Lung adenocarcinoma; ADNOS: Adenocarcinoma not other specified; LUSC: Lung squamous cell carcinoma; THFO: Follicular thyroid cancer; PAAD: Pancreatic adenocarcinoma

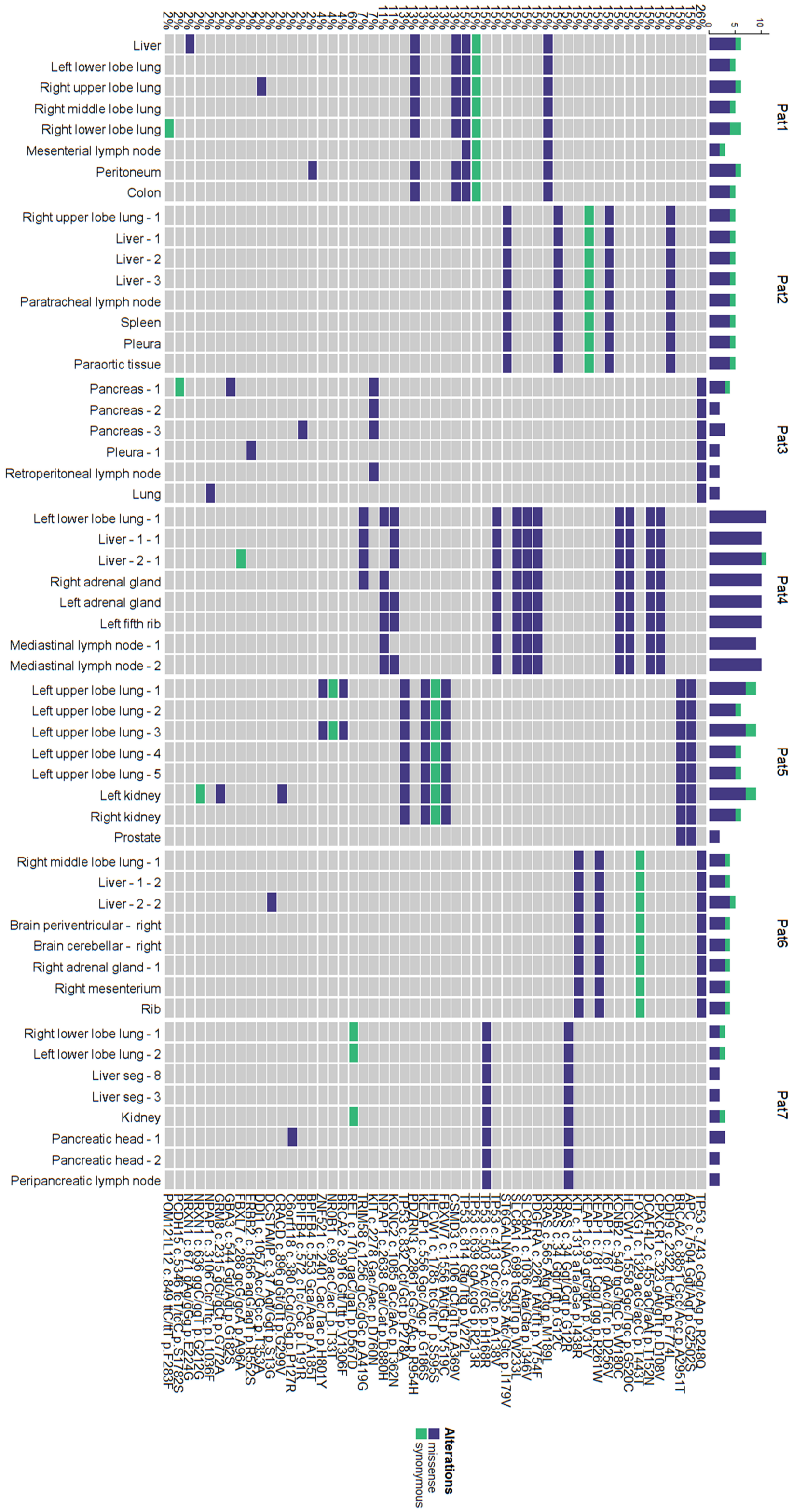

Supplemental Figure S3: Mutational landscape of all tissue biopsies

Oncoprint visualizing the distribution of genetic alterations across lesions of the seven included patients (Pat1 – Pat7). Variants are listed (right) and ordered by their frequency (left). For each lesion, alterations were classified as missense (blue) or synonymous (green). The numbers of respective alterations per lesion are shown as bars above.

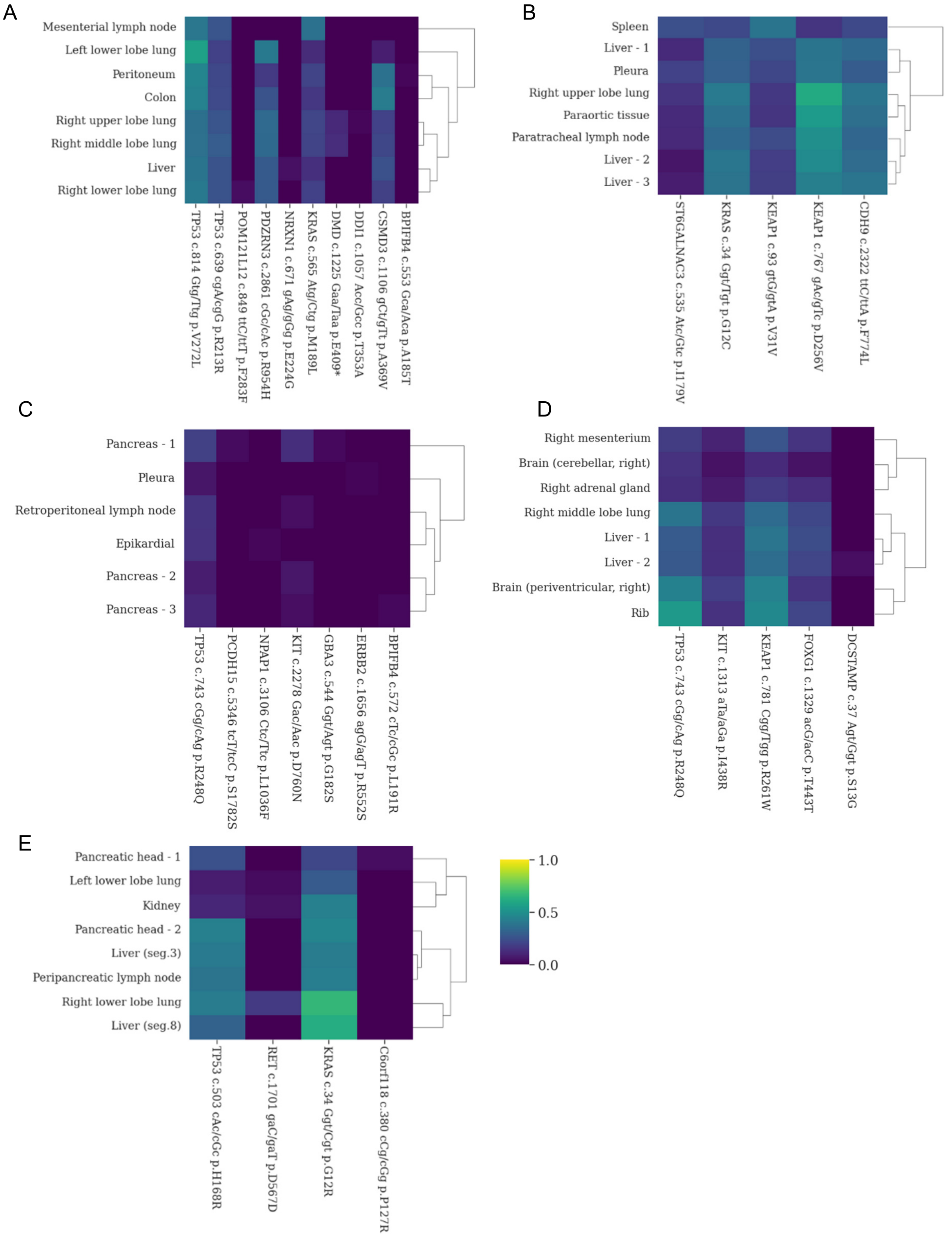

Supplemental Figure S4: Hierarchical clustering of the biopsied tumor lesions (refers to main Figure 1)

Heatmaps of Patient 1 (A), Patient 2 (B), Patient 3 (C), Patient 6 (D), and Patient 7 (E) with hierarchical clustering visualizing the similarity of the lesions in the mutation pattern. Heatmap encodes tissue variant allele frequencies (VAFs) ranging from 0.0 (dark blue) to 1.0 (yellow).

Pat	Gene name	CDS mutation	AA mutation	VAF
1	*SLITRK5*	c.1034 C>A	p.Thr345Asn	0,006
1	*KIT*	c.1478 C>T	p.Ala493Val	0,005
1	*NEUROD4*	c.317 C>T	p.Ala106Val	0,005
1	*NPAP1*	c.1621 T>G	p.Ser541Ala	0,003
1	*DCAF4L2*	c.276 C>T	p.Val92Val	0,002
2	*PDZRN3*	c.2471 C>T	p.Pro824Leu	0,028
2	*GBA3*	c.677 C>T	p.Ala226Val	0,003
3	*SLITRK4*	c.1734 C>A	p.Ile578Ile	0,005
3	*ASTN1*	c.636 C>T	p.His212His	0,003
4	*POM121L12*	c.422 T>C	p.Ile141Thr	0,005
4	*PDZRN3*	c.2532 C>T	p.Asp844Asp	0,004
4	*BRCA1*	c.913 T>C	p.Cys305Arg	0,004
4	*FBXL7*	c.1082 A>T	p.Lys403Ile	0,003
4	*UGT3A2*	c.1408 C>T	p.His470Tyr	0,003
4	*ZNF521*	c.2826 A>G	p.Glu942Glu	0,002
6	*EGFR*	---	---	0,004
7	*USP29*	c.1518 C>G	p.Val506Val	0,003
7	*BRINP2*	c.1563 G>A	p.Gln521Gln	0,003

Supplemental Table S1: Genetic alterations detected exclusively in LBx

List of genetic alterations exclusively detected by LBx, covering gene names, mutations in the coding sequence (CDS), amino acid changes (AA), and their corresponding variant allele frequencies (VAFs) on patient-level (Pat).

Pat	Gene name	CDS mutation	AA mutation	# localizations	VAF (range)
1	*DDI1*	c.1057 A>G	p.T353A	1	0.016
1	*NRXN1*	c.671 A>G	p.E224G	1	0.043
1	*BPIFB4*	c.553 G>A	p.A185T	1	0.018
1	*DMD*	c.1225 G>T	p.E409*	2	0.058–0.246
2	*ST6GALNAC3*	c.535 A>G	p.I179V	8	0.058–0.246
2	*KEAP1*	c.93 G>A	p.V31V	8	0.155–0.385
3	*PCDH15*	c.5346 T>C	p.S1782S	1	0.029
3	*NPAP1*	c.3106 C>T	p.L1036F	1	0.019
3	*ERBB2*	c.1656 G>T	p.R552S	1	0.015
3	*BPIFB4*	c.572 T>G	p.L191R	1	0.022
3	*BPIFB4*	c.572 T>G	p.L191R	1	0.021
3	*GBA3*	c.544 G>A	p.G182S	1	0.086
4	*FBXL7*	c.288 G>A	p.A96A	1	0.055
5	*BRCA2*	c.3916 G>T	p.V1306F	2	0.055–0.061
5	*BRCA2*	c.8851 G>A	p.A2951T	8	0.098–0.592
5	*ZNF521*	c.2401 C>T	p.H801Y	2	0.015–0.057
5	*NRXN1*	c.636 C>T	p.G212G	1	0.214
5	*CRACD*	c.896 G>T	p.G299V	1	0.214
5	*APC*	c.7504 G>A	p.G2502S	8	0.117–0.455
5	*GRM8*	c.2315 G>C	p.G772A	1	0.261
5	*NR0B1*	c.99 C>T	p.T33T	2	0.016–0.089
6	*DCSTAMP*	c.37 A>G	p.S13G	1	0.036
7	*C6orf118*	c.380 C>G	p.P127R	1	0.034

Supplemental Table S2: Genetic alterations detected exclusively in TBx

List of genetic alterations exclusively detected by TBx, covering gene names, mutations in the coding sequence (CDS), amino acid changes (AA), and their corresponding variant allele frequencies (VAFs) on patient-level (Pat). Mutations detected in more than one lesion are combined (# localizations) and provided with a VAF range.
